# Public policies to increase physical activity and reduce sedentary behavior: a narrative synthesis of “reviews of reviews”

**DOI:** 10.1080/16549716.2023.2194715

**Published:** 2023-04-06

**Authors:** Abdullah F. Alghannam, Jesse D. Malkin, Hazzaa M. Al-Hazzaa, Reem AlAhmed, Kelly R. Evenson, Severin Rakic, Reem Alsukait, Christopher H. Herbst, Saleh A. Alqahtani, Eric A. Finkelstein

**Affiliations:** a Lifestyle and Health Research Center, Health Sciences Research Center, Princess Nourah Bint, Abdulrahman University, Riyadh, Saudi Arabia; b Health, Nutrition and Population MENA, World Bank, Washington, DC, USA; c Lifestyle and Health Research Center, King Faisal Specialist Hospital & Research Center, Riyadh, Saudi Arabia; d Department of Epidemiology, Gillings School of Global Public Health, University of North Carolina – Chapel Hill, Chapel Hill, CA, USA; e Health, Nutrition and Population Practice, Middle East and North Africa Region, The World Bank, Washington, DC, USA; f Department of Community Health Sciences, College of Applied Medical Sciences, King Saud University, Riyadh, Saudi Arabia; g Liver Transplant Center, King Faisal Specialist Hospital & Research Center, Riyadh, Saudi Arabia; h Division of Gastroenterology & Hepatology, Johns Hopkins University, Baltimore, MD, USA; i Health Services and System Research Program, Duke-NUS Medical School, Singapore, Singapore; j Human Development Unit, Middle East and North Africa Region, The World Bank, Washington, DC, USA

**Keywords:** Physical inactivity, sedentary behavior, public policy, literature review, public health

## Abstract

**Background:**

Physical inactivity among the general population is of great concern in public health.

**Objective:**

This narrative review aims to identify promising physical activity (PA) public policies based on the best available evidence from the literature.

**Methods:**

The study is a narrative synthesis of ‘reviews of reviews’ of public policies designed to increase physical activity among either (a) youths or (b) the community at large. We searched the literature for reviews of reviews of public policies of any country relevant to physical activity, physical inactivity, or sedentary behaviour published since 1 January 2000, in four databases.

**Results:**

Based on 12 reviews of reviews published between 2011 and 2022, we identified seven potentially effective PA public policies. Six of the seven were youth-based public policies that would be implemented in schools. The seventh was a policy aimed at establishing and promoting walking groups.

**Conclusions:**

Policymakers seeking to increase PA should consider focusing on school-based PA policies and community-based walking groups, as this is where the evidence base is greatest. To implement these policies, pilot studies to assess the efficacy of such programmes in local communities should first be conducted due to methodological limitations in the underlying literature and questions of generalisability and reproducibility.

## Introduction

Amidst soaring rates of obesity [1]] and obesity-related diseases such as type 2 diabetes [2], low levels of physical activity (PA) (any bodily movement produced by skeletal muscles that requires energy expenditure [3]) and high rates of sedentary behaviour (SB) (any waking behaviour characterised by an energy expenditure of 1.5 metabolic equivalents or less while sitting, reclining, or lying [4,5]) have become major concerns in healthcare worldwide. Physical inactivity and SB are independent risk factors for increased morbidity, primarily through rising rates of obesity, chronic disease, and premature mortality [6]. Physical inactivity also raises annual medical expenditures for treating these conditions [7]. For these reasons, governments in many countries are attempting to boost activity levels through public health programmes such as ‘Let’s Move’ in the United States [8], ‘Moving More, Living More’ in the United Kingdom [9], ‘Sport 2030’ in Australia [10], and ‘Sports For All’ in Saudi Arabia [11].

The purpose of this manuscript is to present the results of a narrative synthesis to identify public policies that may be effective in increasing PA and/or reducing SB. We employed this novel strategy – a narrative synthesis of ‘reviews of reviews’ – because it represents an efficient strategy to synthesise this very large literature.

## Methods

### Search strategy

We searched for reviews of reviews of public policies relevant to PA, physical inactivity, or SB published since 1 January 2000. The following search terms were included: ‘physical activ*,’ ‘physical inactiv*,’ ‘sedentary behavior,’ ‘sedentary behaviour,’ ‘review of reviews,’ and ‘review of systematic reviews.’ The databases we searched were PubMed, APA PsycNet, Cochrane Database of Systematic Reviews, and Campbell Collaboration. All searches were conducted during the first week of February 2023.

### Inclusion criteria

For inclusion in this review, articles were required to (1) be a review of reviews (including meta-analyses, narrative reviews, and systematic reviews) focused primarily on interventions aimed at increasing PA, reducing physical inactivity, or reducing SB, (2) have PA or SB outcomes as a major focus, (3) be published in a peer-reviewed journal, (4) provide a detailed description of methods, including a list of the reviews selected for inclusion, (5) primarily address a healthy population rather than those with a specific health condition, (6) be published in English, and (7) not be focused primarily on eHealth, mHealth, primary care, or workplace-based interventions, since such interventions often lie largely outside the purview of public policy. Although some researchers use the terms *policies* and *interventions* synonymously, these are distinct concepts, as has been noted by Rütten et al. (2016) [12] and Gelius et al. (2020) [13]

### Identification of relevant studies

Potentially relevant articles were initially selected by screening titles and abstracts. If abstracts did not provide enough information, the full article was retrieved and screened. Screenings were carried out by two of the study authors (JDM and EAF). Disagreements were resolved through discussion.

### Identification of potentially effective public policy interventions

Public policy has been defined as a ‘course of governmental action established to address the problems of the society at large, rather than individual needs on a smaller scale’ [14]. This is the definition of public policy used in this study. While a large number of reviews have been published within the PA and SB literature, most assessed interventions that have limited relevance to public policy [12]. We reviewed included studies to assess whether interventions were public policy-relevant and summarised the results separately for youth-based and non-youth-based public policies targeting either the general population or a subset of adults. Within these groups, we identified public policies for which evidence of effectiveness was based mainly on trials or natural experiments, as opposed to cross-sectional associations that provide the weakest evidence for causation. We included quantitative estimates of the range of reported effect sizes and described threats to the validity of these results.

## Results

**Figure 1.**
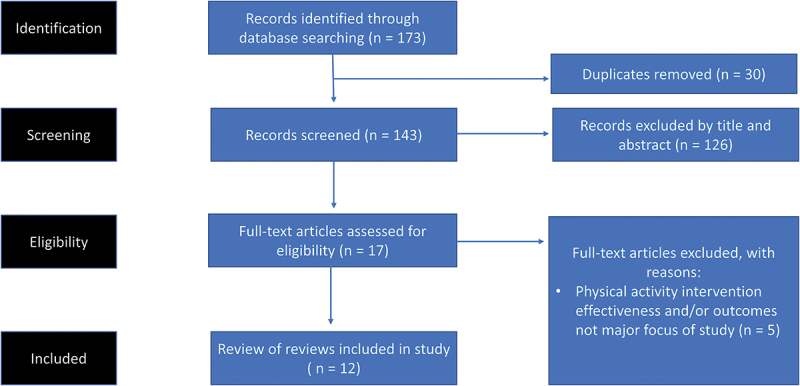
Flowchart of the literature search.

The initial search yielded 173 records. After removing 30 duplicates, we excluded 131 studies that did not meet our inclusion criteria (Figure 1).

This left 12 studies (Table 1), all of were systematic umbrella reviews (systematic reviews of reviews). We discuss these below.

**Table 1.** Reviews of reviews included in study.

Authors	Title of publication	Date of publication	Study objective	Number of reviews included	Quality of reviews assessed?
Kriemler, Meyer, Martin et al. [15]	“Effect of school-based interventions on physical activity and fitness in children and adolescents: a review of reviews and systematic update”	2011	“[T]o summarise recent reviews that aimed to increase PA or fitness in youth and carry out a systematic review of new intervention studies.”	20	Yes. 10-item checklist. Study receives 1 point for each positive item.
van Sluijs, Kriemler, and McMinn [16]	“The effect of community and family interventions on young people’s physical activity levels: a review of reviews and updated systematic review”	2011	A review of reviews and updated systematic review to assess the effect of community and family interventions on the PA levels of young people.	3	Yes. 10-item checklist. Study receives 1 point for each positive item.
Biddle, Petrolini, and Pearson [17]	“Interventions designed to reduce sedentary behaviours in young people: a review of reviews”	2014	“To synthesise systematic reviews and meta-analyses of interventions aimed at decreasing sedentary behaviours among children and adolescents.”	10	No
Brand, Pischke, Steenbock et al. [18]	“What works in community-based interventions promoting physical activity and healthy eating? A review of reviews”	2014	“[T]o assess the effectiveness of community-based interventions to promote physical activity and healthy eating.”	18	Yes. A Measurement Tool to Assess Systematic Reviews (AMSTAR).
Zubala, MacGillivray, Frost et al. [19]	“Promotion of physical activity interventions for community dwelling older adults: A systematic review of reviews”	2017	“To [evaluate] the effects and characteristics of PA promotion interventions aimed at community dwelling people over 50 years old.”	19	Yes. ROBIS.
Belogianni and Baldwin [20]	“Types of Interventions Targeting Dietary, Physical Activity, and Weight-Related Outcomes among University Students: A Systematic Review of Systematic Reviews”	2019	To “identify systematic reviews and meta-analyses of studies aiming to improve health outcomes in university students, to assess their methodological quality, to identify the different types of interventions used and outcomes assessed, and to estimate their overall effect.”	8, of which 2 included PA outcomes	Yes. AMSTAR 2.
dos Santos, Filho, da Silva, et al. [21]	“What works in sedentary behavior interventions for youth: a review of reviews”	2019	To synthesize evidence to “determine which interventions strategies/characteristics are powerful in reducing sedentary behavior among children and adolescents.”	29	Yes. AMSTAR.
Messing, Rütten, Abu-Omar et al. [22]	“How Can Physical Activity Be Promoted Among Children and Adolescents? A Systematic Review of Reviews Across Settings”	2019	To conduct a systematic review of reviews in order to develop national recommendations for PA promotion in Germany.	39	Yes. AGREE.
Gelius, Mesing, Goodwin et al. [13]	“What are effective policies for promoting physical activity? A systematic review of reviews”	2020	To summarize currently available evidence on effective policies for promoting PA	57	No.
Mannocci, D’Egidio, Backhaus et al. [23]	“Are there effective interventions to increase physical activity in children and young people? An umbrella review”	2020	To summarize the existing evidence regarding interventions that aim to increase PA among children and youths.	30	Yes. AMSTAR 2.
Nguyen, Le, Nguyen et al. [24]	“The effectiveness of sedentary behaviour interventions on sitting time and screen time in children and adults: an umbrella review of systematic reviews”	2020	To conduct an umbrella review that synthesizes systematic reviews examining the effectiveness of sedentary behavior interventions on sitting time and screen time.	17	Yes. AMSTAR.
den Braver, Bengoeschea, and Messing et al. [25]	“The impact of mass-media campaigns on physical activity: a review of reviews through a policy lens”	2022	To “summarize the evidence from published reviews on the effectiveness of mass-media campaigns to promote physical activity (PA) or PA-related determinants” [and] “to identify policy-relevant recommendations related to successful PA campaigns.”	22	Yes. AMSTAR 2.

### Youth-based public policies

Ten of the twelve reviews included information on youths or adolescents. Kriemler et al. (2011) [15] performed a review of reviews of school-based PA policies in children and adolescents as well as an updated systematic review. Our focus is on their review of reviews. A total of four reviews were included [16,26–28]. The authors concluded that ‘there is good evidence that school-based interventions can increase PA and fitness in youth’ – at least in the short term [15]. Successful policies included PA sessions during school, activity breaks, modifying playgrounds to make them more PA-friendly, and combining school curriculum and family-based strategies [26]. Effect sizes were not reported. Numerous methodological limitations existed in many of the underlying studies, such as self-reported PA outcomes, lack of blinding, lack of validated PA measures, and lack of studies with long-term follow-up.

van Sluijs et al. (2011) [16] conducted a review of reviews and an update to an earlier review of the effect of community- and family-based interventions on PA in youths and adolescents. The review of reviews included three reviews [16,26,29]. All three concluded that the evidence for policies in most settings was mixed, inconclusive, or ‘not overwhelming.’ A notable exception was school-based policies that included parental/familial involvement. All three reviews reported positive findings. A wide range of such policies were considered. The most successful of these was conducted in Greece in the 1990s [30,31]. However, the results were susceptible to self-report bias, and it is unclear whether these effects translated into actual health benefits or would be replicated in other settings.

Biddle et al. (2014) [17] conducted a review of reviews of interventions designed to reduce SB among young people. Ten reviews were included, of which five were meta-analyses. All reviews reported some level of effectiveness in reducing SB. Electronic television monitors and contingent feedback systems (using television as a reward for PA) were two of the more effective interventions, at least in the short term. However, some evaluated interventions seem to have had limited relevance to government policymaking. Many primary studies cited in these reviews relied on self-reported PA, had small sample sizes, lacked blinding, and excluded long-term follow-up.

Brand et al. (2014) [18] conducted a systematic review of 18 reviews and meta-analyses on the effects of community-based interventions promoting PA and healthy eating. They found weak evidence to support community-based interventions aiming to improve diet and increase PA among children and adolescents. None of the reviews included interventions that were limited to PA, making it impossible to separate the effects of PA interventions from diet interventions. Most of the reviews pertaining to youths and adolescents ‘provided only limited evidence due to a small number of included community-based studies, small sample sizes in the underlying studies, and very few multi-level or environmental change interventions.’

Belogianni and Baldwin (2019) [20] conducted a review of reviews of dietary, PA, and weight-related interventions among university students. Eight reviews were included in the study, but only two [32,68] addressed PA. Both these reviews identified effective interventions. One of the more promising interventions required students to receive exercise instruction and attend three exercise sessions per week [35], while the control group received health instruction. However, the conclusions drawn from these reviews were limited by methodological deficiencies in the primary studies, including self-reported outcomes, attrition, poor reporting, small sample sizes, short durations, and lack of generalisability. Moreover, effect sizes were not reported, and some interventions seemed to lie outside the public policy domain.

dos Santos et al. (2019) [21] conducted a systematic review of randomised controlled trials to determine which interventions are effective in reducing sedentary behaviour among children and adolescents. The study included 29 reviews. Although almost all the reviews reported that interventions were effective, most of them ‘had limited evidence due to the inconsistency of results, low methodological quality and/or small sample size.’ The authors reported low- and moderate-quality evidence that standing desks in classrooms are effective in reducing sedentary behaviour among schoolchildren; however, they concluded that ‘more studies and longer-term trials are needed to determine the efficacy and effectiveness of this approach.’ The authors also cited evidence in support of social support for the family (for example, giving parents materials to facilitate reducing their childrens’ screen time) and electronic monitoring devices. Finally, the authors cited evidence that changes to school curricula that promote physical activity, breaks during class, and educational materials have positive effects in reducing sedentary behaviour. No estimates of effect sizes were reported.

The reviews cited by Messing et al. (2019) [22] found evidence in support of school-based public policies, including short bouts of PA throughout the school day [36], after-school PA programmes [37], providing playground equipment [38,39], providing playground markings [39], and promoting active transport to and from school [40]. However, most of the cited reviews did not report effect sizes, and many studies relied on self-reported PA. Where effect sizes were reported, they were based on a vast array of policies, making it difficult to isolate the effects of any given policy (see, e.g., [41] and [42]). Moreover, some interventions, like changes in the home to limit screen time, seem to lie largely outside the public policy domain [22]. As a result, this review of reviews is of limited usefulness for public policy purposes.

Based on their analysis of 57 reviews, Gelius and colleagues (2020) [13] recommended several school-based public policies: mandatory physical education lessons, after-school sports programmes, classroom activity breaks, longer recesses, and PA-friendly playgrounds. The estimated effect size of such policies varied widely, ranging from an increase in moderate-to-vigorous PA (MVPA) per day of 3 min [43] to more than an hour (the combined effect of all the school-based policies assessed by Bassett et al. [2013] [44]). These estimates, however, relied on self-reported PA. The authors also recommended community-wide mass media campaigns targeting children. However, they cited only one review by Pate et al. (2011) [45] to support their recommendation. Pate and colleagues, in turn, cited only two primary studies [33,34] on the effects of mass media and advertising. Both relied on self-reported PA only and neither used an experimental design. Reverse causation (the possibility that physically active children noticed the mass media campaign more than inactive children) and other sources of bias could not be ruled out.

Mannocci et al. (2020) [23] conducted a systematic review of systematic reviews and meta-analyses to summarise existing evidence on interventions designed to increase PA in youths. A total of 30 reviews were included in the study. Most interventions were conducted in educational settings such as primary schools, secondary schools, and universities. Unfortunately, the authors’ ability to draw conclusions was limited because ‘either the quality of the systematic review or meta-analysis was poor or because, as noted in several reviews, the quality of primary studies was poor.’ More than two-thirds of the included studies were classified as low or very low methodological quality. While drawing firm conclusions was not feasible, some interventions seemed more promising than others. For example, school-based interventions that involved family members and/or teachers seemed to be more effective than those that did not. Multicomponent approaches such as programmes combining diet and PA programming appeared to be more effective than single-component interventions.

Nguyen et al. (2020) [24] conducted an umbrella review to assess evidence on interventions designed to reduce SB in the general population among all age groups and settings. The study included 17 meta-analyses. Most articles primarily addressed workplace and/or primary care interventions, which are beyond the scope of our review due to their limited public policy relevance. We focus on the results of six meta-analyses that addressed a wide range of policy interventions targeted at children and adolescents, many of which were implemented in schools [41,42,46–49]. Overall, most of the reviews supported the effectiveness of interventions designed to reduce sedentary time or specifically screen time in children and adolescents. Two meta-analyses reported reductions in SB of about 18 min per day [42,47]. Four of the six meta-analyses [41,42,46,48] were characterised as low quality. In the two meta-analyses rated as moderate quality [47,49], the results were mixed. Those two reviews included trials in which SB outcomes were self-reported rather than based on objective data. Ultimately, this review of reviews is of limited use for public policymaking because the effect size estimates are based on dozens of different interventions, making it impossible to isolate the effect of any specific intervention [24]. Moreover, many interventions – e.g. cognitive and behavioural training and changes in the home to limit screen time – arguably have limited public policy relevance (i.e. they are interventions that typically would be implemented by individuals or families, not governments).

### Non-youth-based public policies

Four of the twelve reviews in our analysis included information on non-youth interventions with potential relevance to public policy. Brand et al. (2014) [18] reported mixed evidence on community-based PA interventions targeting adults. The most promising PA intervention was walking groups. One review reported that the most successful walking group interventions can increase walking by ‘up to 30–60 min a week on average, at least in the short term’ [50]. Another review concluded that such interventions have a medium-sized effect on PA but noted significant methodological limitations in the literature, such as the small number of studies using objective measures of PA [51]. Many interventions in these reviews (e.g. discussion groups) arguably lie outside the public policy domain, limiting the relevance of such studies for policymaking purposes. Walking groups have been supported by at least one government [52] but can also be organised through non-governmental means.

Zubala et al. (2017) [19] performed a systematic review of reviews to assess the effectiveness of PA interventions targeting adults aged over 50. A total of 19 reviews met the authors’ inclusion criteria. Most reviews reported an increase in PA levels due to interventions. Chase (2015) [53], for example, reported an overall mean effect size of 620 steps per day or 73 min of PA per week. Hobbs et al. (2013) [54] reported an overall mean effect size of 2,197 steps per day. However, only 3 [55–57] of the 19 reviews cited by Zubala and colleagues were judged to have a low risk of bias. This ‘unavoidably impacts on the quality of evidence summarized in the current review, which should be interpreted with caution,’ the authors wrote. The three low-bias studies all reported only small increases in PA. Most of the included reviews consisted of studies that relied on self-reported PA outcomes. Finally, many primary studies in this literature had durations of one year or less. Zubala and colleagues concluded that ‘ways to ensure effective maintenance beyond one year are unclear’ [19]. As in the paper by Nguyen and colleagues [24], all of the reviews cited by Zubala and colleagues covered a vast array of interventions, making it difficult to isolate the effects of any one intervention. Moreover, many of the reviewed interventions (e.g. motivational counselling, goal setting, problem-solving, behavioural goal setting, and self-monitoring of behaviour) seemed to lie outside the realm of public policy. These factors limit the usefulness of this literature for identifying appropriate interventions for consideration by governments.

Gelius and colleagues (2020) [13] recommended infrastructure, urban design, and ‘built environment’ policies. They cited nine reviews [58–66], all of which we were able to locate. One of the studies [58] did not report the effects of these policies on PA. Most of the remaining studies [59–64,65] relied heavily or exclusively on cross-sectional analyses, with no possibility of drawing causal inferences. Several reviews concluded that certain policies designed to increase walking and cycling (e.g. dedicated bike paths, pro-dog walking policies) may have positive effects [59,61–63], but the size of such effects was either not reported, unknown, ‘difficult to isolate,’ or ‘contentious’ [58,59,61,64] One of the cited reviews [63] relied exclusively on cross-sectional analyses, which cannot prove causality. Two reviews reported a lack of high-quality evidence in support of various policies designed to increase walking and cycling (e.g. road/sidewalk safety measures, providing written information, provision of a bicycle, provision of self-help materials to people considering active commuting and irregular commuters, meetings with physicians, PA prescriptions, and group counselling) [60,65]. An unstructured review that was published in a book cited evidence in support of policies to promote walking [66]. It is unclear whether this review was peer reviewed.

den Braver et al. (2022) conducted a review of reviews to (1) summarise the evidence on the effectiveness of mass media campaigns designed to promote PA and (2) identify policy-relevant recommendations. The study included 22 reviews that examined PA-related proximal outcomes (i.e. awareness, recall of messages), intermediate outcomes (i.e. changes in knowledge, awareness, and/or attitudes), and distal outcomes (i.e. changes in PA). This literature reported positive effects of mass media campaigns on proximal outcomes, modest but usually positive effects on intermediate outcomes, and mixed evidence regarding distal outcomes. ‘Campaigns that focused on social norms (rather than risk messaging) were found to be most effective, as were those targeting specific PA behaviours (such as walking),’ the authors concluded (citations omitted). Many of the primary studies were of short duration and/or relied on self-reported PA measures, limiting their definitiveness. No quantitative estimates of effect size were provided. With respect to public policy, the authors recommend mass media initiatives that are (a) long-term, (b) incorporated into broader community interventions, and (c) tailored to specific subpopulations such as low socioeconomic groups.

### Potentially effective policies

In Table 2, we tabulate a list of well defined and potentially effective public policies for which quantitative estimates of effect size exist. Six of the seven policy interventions are youth-based policies that would be implemented in schools. The remaining one is a policy aimed at establishing and promoting walking groups.

**Table 2.** Potentially effective PA-promoting public policies for which quantitative estimates are available.

Public policy	Description	Reported effect size	Source
**Physical education**			
**•** Mandatory PE	a policymandating daily physical education for all schoolchildren	23 minutes of MVPA gained per day	Bassett, Fitzhugh, Heath et al. (2013) [44]
**•** Standardised PE curricula	Standardized curricula (e.g. Sports, Play, and Active Recreation for Kids [SPARK]) that increase the proportion of MVPA time during physical Education.	6 minutes of MVPA gained per day	Bassett, Fitzhugh, Heath et al. (2013) [44]
**Breaks during school**			
**•** Recess	Recess periods of 15 minutes for elementary and middle school children.	7 minutes of MVPA gained per day	Bassett, Fitzhugh, Heath et al. (2013) [44]
**•** Classroom activity breaks	Breaks – usually 10 minutes in duration – that add a physically active component to the academic material being taught. These programs typically are offered in elementary schools (see, e.g. Physical Activity Across the Curriculum, TAKE 10! and Energizers).	19 minutes of MVPA gained per day	Bassett, Fitzhugh, Heath et al. (2013) [44]
**School programming**			
**•** After-school activity programs	Provision of a supervised PA program for children for several hours between the end of the school day and the time parents pick up their children.	22.6% increase in weekly MVPA (from a baseline of 1,902.7 metabolic equivalents)	Kristensen, Flottemesch, Maciosek et al. (2014) [67]
		10 minutes of MVPA gained per day	Bassett, Fitzhugh, Heath et al. (2013) [44]
**Other**			
**•** Walking groups	e.g., lay-mentored meetings, led walking training, and educational sessions	30–60 minutes per week	Ogilvie, Foster, Rothnie et al. (2007) [50]

We define “potentially effective” policies as specific public policies deemed effective in reviews based on trials or natural experiments. All of these estimates are based on studies with major methodological limitations. These estimates may be upwardly biased due to the lack of blinding and overreporting of physical activity.

Abbreviations: MVPA: moderate to vigorous-intensity physical activity; PE: physical education.

## Discussion

The aim of this narrative review was to provide an overview of the evidence on PA-promoting public policies. This review identified numerous shortcomings in the literature, such as reliance on subjective measures of PA [16,27,28,41–44,46,47,67–71] and short study durations [15,17,19,20]. Self-reported PA data are likely to be upwardly biased due to the well-documented tendency of people to overreport their own PA [72–74] and evidence that interventions can induce over-reporting [75]. Other threats to validity included high attrition, poor reporting, small sample sizes, and lack of generalisability.

Despite these concerns, the literature we reviewed provides some evidence to support school-based policies that promote PA among children and adolescents [13,15,16,21–23]. This includes mandatory physical education, standardised physical education curricula, longer recesses, classroom activity breaks, after-school PA programmes, and the provision of playground equipment that encourages PA. We also found evidence to support community-based walking groups and walking/bike paths [13,18], although, for the latter, no quantitative estimates of effect size were reported. Our findings are largely consistent with those of The Community Guide, a US Centers for Disease Control and Prevention task force that has conducted numerous literature reviews to assess PA-promoting policies [76–78]. Other PA interventions that show promise (such as using television as a reward for PA) are of limited direct relevance for government policymaking, aside from educating the public about potential benefits.

Our review focused on identifying potentially effective programmes. There is additional literature focusing on cost-effectiveness. Two reviews [79,80] and one review of reviews [81] reported that school-based physical activity policies are among the most cost-effective PA policies. This finding bolsters the case for consideration of these interventions. Abu-Omar and colleagues [81] further report that environmental approaches may be cost-effective but state that the underlying evidence of effectiveness is inconsistent.

### Strengths and limitations

Our review considers information from a substantial number of published, peer-reviewed reviews over a long period of time. However, the study is subject to several limitations. First, our study included reviews of reviews published between 2011 and 2022. Due to lag time in publication, the most recent of these studies searched for reviews only through 1 March 2021. It is possible that more recent studies would produce additional information. Second, we excluded several categories of interventions – e-health, mobile health, primary health, and workplace – to focus on public policies most likely to be considered for implementation by government policymakers. This is similar to the approach used by Gelius et al. [13] Many such interventions may be effective, but their public policy relevance seems to be limited. Third, our reliance on reviews of reviews rather than primary studies could lead to incorrect conclusions if there is publication bias or other biases in the reviews we relied upon. Moreover, databases other than the ones we searched and the gray literature could yield further insight but were not included here. Fourth, the vast majority of studies in the literature were conducted in single locations in Western countries (e.g., [16]). It should not be assumed that the results of such studies can be generalised to other settings where climate, topography, attitudes, income, and other factors related to PA are likely to differ [82]. Fifth, we did not independently assess the quality of the reviews of reviews included in this analysis. Sixth, we relied only on English-language reviews, potentially missing other important reviews. Future reviews should address these limitations as well as the feasibility of scaling up evidence-based interventions.

## Conclusion

In this narrative review, we assessed the evidence of the effectiveness of a wide array of PA public policies. We identified seven PA policy-relevant public policies that have been deemed effective in one or more reviews of reviews and for which quantitative estimates of effect size exist. Six of these interventions are school-based PA policies, and the seventh is community-based walking groups. These policies should be considered for adoption by policymakers who would like to increase population levels of PA. However, prior to making a significant investment in any proposed public policies, we recommend pilot studies to evaluate their effectiveness in the local setting due to methodological limitations in much of the underlying literature and concerns regarding reproducibility, generalisability, and scalability.

## Paper context

Our narrative review aims to identify promising physical activity public policies based on the best available evidence from the literature. Our findings suggest that policymakers should consider school-based physical activity programmes and community-based walking groups to increase physical activity levels. However, before making a significant investment in public policies, we recommend that governments conduct pilot studies to assess the efficacy of such programmes due to methodological limitations in the underlying literature.
